# Potential drug–drug interactions in patients with cardiovascular diseases: findings from a prospective observational study

**DOI:** 10.1186/s40545-021-00348-1

**Published:** 2021-07-26

**Authors:** Zarka Akbar, Sundas Rehman, Asad Khan, Amjad Khan, Muhammad Atif, Nafees Ahmad

**Affiliations:** 1 Department of Pharmacy Practice, Faculty of Pharmacy and Health Sciences, University of Balochistan, Quetta, Pakistan; 2 Department of Pharmacy, Quaid-i-Azam University, Islamabad, Pakistan; 3 Department of Pharmacy Practice, Faculty of Pharmacy, The Islamia University of Bahawalpur, Bahawalpur, Pakistan

**Keywords:** CVD, Lexicomp, Myocardial infarction, Pakistan, Potential drug–drug interactions

## Abstract

**Background:**

Patients with cardiovascular diseases (CVD) are at high risk of experiencing drug–drug interactions (DDIs). The objective of this study was to evaluate the frequency, level and risk factors associated with potential-DDIs (pDDIs) in hospitalized CVD patients at cardiology departments of two tertiary care hospitals in Quetta, Pakistan.

**Methods:**

In the current prospective observational study, a total of 300 eligible CVD inpatients were evaluated for pDDIs using Lexicomp Interact®. The pDDIs were classified into class A (no known interaction); B (no action needed); C (monitor therapy: it is documented that the benefits of an interaction outweigh the risk, appropriately monitor therapy in order to avoid potential adverse outcomes); D (consider therapy modification: it is documented that proper actions must be taken to reduce the toxicity resulting from an interaction); X (avoid combination: the risk of an interaction outweighs the benefits and are usually contraindicated). Multivariate binary logistic regression analysis was used to find factors associated with the presence of Class-D and/or X pDDIs. A *p*-value < 0.05 was considered statistically significant.

**Results:**

With a median of 8.50 pDDIs per patient, all patients (100%) had ≥ 1 pDDIs. Out of total 2787 pDDIs observed, 74.06% (*n* = 2064) were of moderate and (*n* = 483) 17.33% of major severity. Class C pDDIs were most common (*n* = 1971, 70.72%) followed by D (*n* = 582, 20.88%), B (*n* = 204, 7.32%) and X (*n* = 30, 1.08%). Suffering from cardiovascular diseases other than myocardial infarction (OR 0.053, *p*-value < 0.001) and receiving > 12 drugs (OR 4.187, *p*-value = 0.009) had statistical significant association with the presence of class D and/or X pDDIs.

**Conclusion:**

In the current study, pDDIs were highly prevalent. The inclusion of DDI screening tools, availability of clinical pharmacists and paying special attention to the high-risk patients may reduce the frequency of pDDIs at the study sites.

## Background

By causing an estimated 17.9 million deaths annually, cardiovascular diseases (CVDs) are the leading lethal diseases globally, making 31% of all-cause mortality [1]. Because of multiple etiologies and concurrent comorbidities, CVD patients are treated with a complex therapeutic regimen comprising multiple different drugs. For example, in the United States of America, the elderly CVD patients (age > 65 years) had eight concurrent comorbidities and took 13 medications on average [2]. Likewise, prescription of a large number of different drugs (range 2–24 drugs) to CVD patients have been reported by studies conducted elsewhere [3–9].The presence of multiple etiologies, concurrent comorbidities, complex medication regimen and the type of drugs CVD patients receive make them a high-risk group for the incidence of drug–drug interaction (DDI) defined as “alteration in a drug (object drug) effect caused by the concurrent administration of another drug (precipitant)” [3–8]. In an actual DDI, clinically meaningful change in the effect of object drug consequently leads to either a harmful or beneficial outcome [7]. The presence of harmful DDIs not only interferes with the desired goal of therapy, but also results in increased rate of morbidity, mortality and health care costs [3–8, 10]. Previously published studies have reported that 17–27% of complications in hospitalized patients were due to DDIs [11, 12]. In a Serbian study, 9.69% of hospital admissions were attributed to DDIs [4]. On the other hand, a potential DDI (pDDI) occurs when two drugs known to interact are concomitantly administered, irrespective of whether a clinically meaningful consequence occurs or not, and they essentially occur prior to actual DDIs [13]. The previously reported prevalence of pDDIs among CVD patients ranges from 21.3 to 96.9% [3–8, 14–16]. As in the published literature, pDDIs are acknowledged the predictable and avoidable causes of adverse drug events (ADEs); therefore, the current standards in research and clinical practice focus and emphasize on recognizing and implementing actions to avoid pDDIs and hence preventable ADEs.

Unfortunately, with an estimated prevalence of 17.5%, Pakistan is a high burden CVD country where 29% of all-cause mortality is attributed to CVD [17]. Very few studies have evaluated the frequency, severity and risk factors associated with pDDIs among CVD patients in Pakistan. In these studies, the reported prevalence of pDDIs ranged from 42 to 96.5% [3, 6, 14, 18]. A study conducted among hospitalized CVD patients at Ayub Teaching Hospital, Abbottabad, Pakistan, has reported that 91.6% patients had at least one pDDI [3]. Out of the total 5109 pDDIs observed, most (55%) were of moderate or major (45%) severity, and the variables of patients’ age of ≥ 60 years, hospital stay ≥ 7 days and taking ≥ 7 drugs had statistical significant associations with the incidence of pDDIs [3]. Similar high prevalence of pDDIs (96.5%) was observed among CVD patients who received treatment at intensive care units (ICU) of Khyber Teaching Hospital (KTH) and Hayatabad Medical Complex (HMC), Peshawar, Pakistan [6]. In the said study, taking ≥ 3 drugs emerged as the only risk factor for the incidence of pDDIs at both KTH and HMC [6].

Information about pDDIs in any clinical setting has the potential to help the clinicians to limit the patient’s exposure to ADEs and improve their therapeutic outcomes. Variation in the reported prevalence of pDDIs due to using different screening tools, methodologies, settings and prescribing patterns advocates that it is important to evaluate the incidence of pDDIs in different clinical settings, so that the evidence can be generated, aggregated and summarized in each country. Furthermore, interventions designed for reducing the frequency of pDDIs in healthcare settings of a country are likely to be more effective, if prior to their development, the incidence, pattern and risk factors of pDDIs are determined accurately. However, to the best of our knowledge there is a complete lack of published information about pDDIs in CVD patients from Balochistan, which is by area the largest province of Pakistan. Therefore, the current study was conducted with the objective to provide information about the frequency, level and risk factors associated with pDDIs in hospitalized CVD patients treated at two tertiary care hospitals in Quetta, Balochistan.

## Methods

### Study design and study setting

This was a prospective observational study carried out between 1st October 2020 and 31st December 2020 at the cardiology departments of Bolan Medical Complex Hospital (BMCH) and Sandeman Provincial Hospital (SPH) Quetta, Balochistan. The BMCH and SPH are major public tertiary care teaching hospitals located in the capital of the province (Quetta city) and provide health care services to 2.2 million people of Quetta. Additionally, being the major public tertiary care teaching hospitals in Balochistan, both hospitals have the wide catchment area of the whole province and the nearby border areas of Afghanistan and Iran. In 2018, approximately 515, 953 patients had been examined in out-patient departments of BMCH and 23,418 patients were treated as inpatients [19]; whereas, SPH is an 800-bed hospital which provides health services to an overwhelming 8000 to 10,000 patients every day [20].

### Study subjects

All CVD patients who were at least 18 years old, received at least two drugs, treated as inpatients for at least 24 h at the cardiology wards or cardiac intensive care units (CICUs) of the study sites and who themselves or their treatment supporters were willing to participate in the study by giving written or oral consent (in case of illiterate patients or their treatment supporters) were included in the study and followed until their discharge from the hospitals. Non-probability convenient sampling technique was used for enrolling the eligible study participants. Sample size was calculated by using Daniel’s sample size calculation formula, i.e., *Z*^2^*P* (1 − *P*)/*d*^2^ [21], where *n* = required sample size, *Z* = *Z*-statistics for a level of confidence (for 95% level of confidence, *Z* = 1.96), *P* = expected prevalence or proportion of the condition in population based on previous published studies or pilot studies [in proportion of 1, if 20%, *p* = 0.2. So, the estimated frequency of pDDIs in CVD inpatients was 91.6% or 0.916 [3], *d* = absolute error or precision (in proportion of 1, if 5%, *d* = 0.05). So, by putting these values in the above-mentioned formula, the sample size was ought to be 120 patients per hospital. Those patients who were visiting the cardiology departments as outpatients and who stayed in the cardiology wards for less than 24 h were excluded from the study.

### Data collection

A purpose-designed data collection form based on the previously published studies and suggestions of the clinicians at the study sites was used to collect the data. The data collection form contained variables regarding patient’s socio-demographics, clinical, laboratory and medication data. The patients’ main diagnosis and comorbidities present were recorded on the basis of information documented in their medical charts. The medicines prescribed to the patients were recorded by their generic names and classified under their respective pharmacological classes. Each active moiety of a fixed-dose combination was recorded as an individual drug.

### Screening of potential drug–drug interactions

Lexicomp Interact® [22] was used for screening pDDIs. It is a widely used, highly sensitive and specific DDIs screening tool [5, 23–25]. Lexicomp Interact® classifies pDDIs on the basis of severity into minor (inconvenient interaction or little effect), moderate (deterioration of patient’s condition) and major (life threatening or causing permanent damage). Based on the level of urgency and timely response towards these interactions, a risk rating category is given in Lexicomp interact®. This included class A (no known interaction), B (no action needed), C (monitor therapy: it is documented that the benefits of an interaction outweigh the risk, appropriately monitor therapy in order to avoid potential adverse outcomes), D (consider therapy modification: it is documented that proper actions must be taken to reduce the toxicity resulting from an interaction), X (avoid combination: the risk of an interaction outweighs the benefits and are usually contraindicated) [22].

### Statistical analysis

Data were analyzed using SPSS (version 23). Frequencies and percentages were used for displaying categorical data, whereas continuous data were presented as mean ± standard deviations, median and ranges. Univariate binary logistic regression analysis was used to find association between patient’s characteristics and the presence of D and/or X pDDIs. In order to get the final variables associated with the presence of D and/or X pDDIs, all those variables which had a *p*-value < 0.2 in univariate analysis were checked for collinearity and entered in multivariate binary logistic regression (MVBLR) analysis. A *p*-value < 0.05 was considered statistically significant.

## Results

### Sociodemographic and clinical characteristics of patients

The sociodemographic and clinical characteristics of the study participants are presented in Table 1. A total of 300 CVD patients were included in the study, 150 from each of the two hospitals. The patients had a mean age of 57.80 ± 15.90 years. The majority of patients were males (*n* = 168, 56%), ≥ 60 years old (*n* = 117, 39%), non-smokers (*n* = 177, 59%), suffered from ST-elevated myocardial infarction (STEMI) (*n* = 123, 41%) and had ≥ 1 comorbidity (*n* = 180, 60%). The patients stayed in hospital for a mean 4.45 ± 2.40 (range 2–14) days and received 10.21 ± 2.6 drugs (range 6–21).

**Table 1:** Sociodemographic and clinical characteristics of study participants

Variables	No. (%)/Mean ± SD/median (range)
Gender	
Female	132 (44.0)
Male	168 (56.0)
Age (years)	
18–30	18 (6.0)
31–40	39 (13.0)
41–50	54 (18.0)
51–60	72 (24.0)
> 60	117 (39.0)
Hospital	
BMCH	150 (50.0)
SPH	150 (50.0)
Residence	
Rural	141 (47.0)
Urban	159 (53.0)
Nationality	
Pakistani	267 (89.0)
Afghan	33 (11.0)
Smoking	
Non-smokers	177 (59.0)
Ex-smokers	24 (8.0)
Active smokers	99 (33.0)
Type of CVD	
STEMI	123 (41.0)
NSTEMI	33 (11.0)
CAD	21 (7.0)
Cardiomyopathy	69 (23.0)
Valvular heart disease	12 (4.0)
Heart failure	24 (8.0)
Cardiac arrhythmias	18(6.0)
Comorbidity	
No	120 (40.0)
Yes	180 (60.0)
Number of comorbidities	1 (0–3)
0	120 (40.0)
1	96 (32.0)
2	66 (22.0)
3	18 (6.0)
Types of comorbidity	
Hypertension	116
Diabetes mellitus	78
Anemia	22
Others	45
Length of hospital stay (days)	4.45 ± 2.40 (2–14)
≤ 3	(49.0)
4–7	(42.0)
> 7	(9.0)
Number of drugs prescribed	10.21 ± 2.6 (6–21)
6–9	135 (45.0)
10–12	114 (38.0)
> 12	57 (17.0)

*CAD* coronary artery disease; *CVD* cardiovascular disease; *NSTEMI* non-ST elevated myocardial infarction; *SD* standard deviation; *STEMI* ST-elevated myocardial infarction

### Frequency and classification of pDDIs

In this study, all the 300 patients (100%) had ≥ 1 pDDI. A total of 2787 pDDIs were observed with a median of 8.50 pDDIs per patient (range 2–19). Out of 2787 pDDIs, 2064 (74.06%) were of moderate severity followed 483 (17.33%) major and 240 (8.61%) minor pDDIs. Lexicomp’s reliability rating was poor for 21 (0.75%), fair for 2163 (77.61%), good for 498 (17.87%) and excellent for 105 (3.77%) pDDIs. On the basis of Lexicomp’s risk classification, class C (monitor therapy) was the most common (*n* = 1971, 70.72%) class followed by class D (consider therapy modification) (*n* = 582, 20.88%), class B (no action needed) (*n* = 204, 7.32%), and class X (avoid combination) (*n* = 30, 1.08%) (Table 2). The pairs of drugs involved in class D and X pDDIs and their potential consequences are given in Table 3.

**Table 2:** Classification of pDDIs

Classification	pDDI No.(%)
Risk rating	
A	**–**
B	204 (7.32)
C	1971 (70.72)
D	582 (20.88)
X	30 (1.08)
Severity	
Minor	240 (8.61)
Moderate	2064 (74.06)
Major	483 (17.33)
Reliability	
Poor	21 (0.75)
Fair	2163 (77.61)
Good	498 (17.87)
Excellent	105 (3.77)

**Table 3:** Drug pairs involved in class X and D pDDIs and their potential consequences

Category	Drug interacting pair	Frequency	Potential consequence
X	Albuterol + carvedilol	15	Diminished bronchodilatory effects of albuterol
	Alprazolam + orphenadrine	3	Increased CNS depressant effects of orphenadrine
	Clarithromycin + ivabradine	3	Increased risk of ivabradine toxicity
	enoxaparin + rivaroxaban	3	Increased risk of bleeding
	Orphenadrine + dimenhydrinate	3	Increased potential for CNS depression
	tramadol + nalbuphine	3	May diminish the analgesic effect of tramadol
D	Aspirin + enoxaparin	229	Increased risk of bleeding
	Clopidogrel + enoxaparin	214	Increased risk of bleeding
	Clopidogrel + omeprazole	94	Decreased clopidogrel effectiveness and therapeutic failure
	Clopidogrel + esomeprazole	6	Decreased clopidogrel effectiveness and therapeutic failure
	Aspirin + rivaroxaban	6	Increased risk of bleeding
	Insulin + sitagliptin	6	Increased risk of hypoglycemia
	Atorvastatin + diltiazem	3	Increased risk of myositis, rhabdomyolysis and hepatotoxicity
	Atorvastatin + clarithromycin	3	Increased serum concentration of atorvastatin may lead to rhabdomyolysis
	Clarithromycin + verapamil	3	Decreased metabolism and increased therapeutic effects of verapamil
	Warfarin + amiodarone	3	Increased risk of bleeding
	Warfarin + meloxicam	3	Increased risk of bleeding
	Digoxin + amiodarone	3	Increased concentration of digoxin and possible toxicity
	Domperidone + escitalopram	3	Increased risk of QT interval prolongation
	Furosemide + meloxicam	3	May decrease the diuretic effect of furosemide
	Tramadol + dimenhydrinate	3	Increased CNS depressant effect of tramadol

### Factors associated with the presence of class D and/or X pDDIs

A total of 240 (80%) patients have ≥ 1 class D and/or X pDDIs. Among the 240 patients who had D and/or X pDDIs, 42 (17.5%) patients had one, 66 (27.5%) had two, 87 (36.2%) had three and 45 (18.75%) had four D and/or X pDDIs. In MVBLR analysis, the final factors which had statistically significant association with the presence of class D and/or X pDDIs were suffering from CVD other than myocardial infarction (MI), i.e., STEMI and Non-STEMI (OR 0.053, *p*-value < 0.001) and receiving > 12 drugs (OR 4.187, *p*-value = 0.009). This model was based on a non-significant Hosmer–Lemeshow test (*p*-value = 0.376) and overall percentage of 84% from classification table. This implies that the prevalence of class D and/or X pDDIs was significantly lower in those patients who suffered from CVD other than myocardial infarction (STEMI and non-STEMI) and significantly higher in those who received > 12 drugs (Table 4).

**Table 4:** Factors associated with the presence of class D and/or X pDDIs

Variables	pDDIs (D and/or X)	Univariate analysis		Multivariate analysis	
	No. (%)				
		OR (95%CI)	*p*-value	OR (95%CI)	*p*-value
Gender					
Female	102 (77.3)	Referent			
Male	138 (82.1)	1.353 (0.767–2.385)	0.296		
Age (years)					
< 60	147 (80.2)	Referent			
≥ 60	93 (79.5)	0.949 (0.532–1.692)	0.859		
Hospital					
BMCH	123 (82.0)	Referent			
SPH	117 (78.0)	0.737 (0.432–1.418)	0.237		
Residence					
Rural	117 (83.0)	Referent			
Urban	123 (77.4)	0.701 (0.394–1.246)	0.226		
Nationality					
Pakistani	210 (78.7)	Referent		Referent	
Afghan	30 (90.9)	2.714 (0.799–9.216)	0.109	2.337 (0.604–9.042)	0.219
Smoking					
Non-smokers	135 (76.3)	Referent		Referent	
Ex-smokers	21 (87.5)	2.178 (0.619–1.755)	0.225	2.435 (0.618–9.591)	0.203
Active smokers	84 (84.8)	1.742 (0.910–3.335)	0.094	1.757 (0.831–3.714)	0.14
Type of CVD					
STEMI + NSTEMI	150 (96.2)	Referent		Referent	
Others	90 (62.5)	0.067 (0.028–0.161)	< 0.001	0.053 (0.021–0.132)	< 0.001
Comorbidity					
No	96 (80.0)	Referent			
Yes	144 (80.0)	1.000 (0.561–1.782)	1		
Number of comorbidities					
0	96 (80.0)	Referent			
1	78 (81.3)	1.083 (0.549–2.139)	0.818		
2	51 (77.3)	0.850 (0.410–1.762)	0.662		
3	15 (83.3)	1.250 (0.335–4.669)	0.74		
Hypertension					
No	145 (78.8)	Referent			
Yes	95 (81.9)	1.217 (0.674–2.195)	0.515		
Diabetes mellitus					
No	177 (79.7)	Referent			
Yes	63 (52.6)	1.068 (0.557–2.048)	0.844		
Anemia					
No	224 (80.6)	Referent			
Yes	16 (72.7)	0.643 (0.240–1.720)	0.379		
Other comorbidities					
No	207 (81.2)	Referent			
Yes	33 (73.3)	0.638 (0.307–1.325)	0.228		
Length of hospital stay (days)					
≤ 3	120 (81.6)	Referent			
04-Jul	96 (76.2)	0.720 (0.401–1.293)	0.271		
> 7	24 (88.9)	1.800 (0.505–6.414)	0.365		
Number of drugs prescribed					
6–9	105 (77.8)	Referent		Referent	
10–12	90 (78.9)	1.071 (0.584–1.964)	0.823	1.674 (0.818–3.427)	0.159
> 12	45 (88.2)	2.143 (0.834–5.505)	0.113	4.187 (1.427–12.285)	0.009

*CI* confidence interval; *CVD* cardiovascular disease; *NSTEMI* non-ST elevated myocardial infarction, *pDDI* potential drug–drug interaction; *OR* odds ratio; *STEMI* ST-elevated myocardial infarction

## Discussion

To the best of our knowledge, this is the first study to evaluate the frequency, level and risk factors associated with pDDIs in CVD patients at two tertiary care hospitals in Balochistan, Pakistan. The studied patients had a mean age of 57.80 ± 15.90 years, majority of them were males (56%) and had MI as the main diagnosis (52%). The predominance of males and MI as the most common type of CVD in the current cohort was in compliance with the reported global epidemiology of CVD [25] and other studies from Pakistan [3, 6, 14]. In our study, the frequency of pDDIs (100%) in CVD patients was significantly higher than reported by studies conducted in India (30.67%) [15], Iran (43.4%) [16], Nepal (62.5%) [8], Ethiopia (74.4%) [7] and Serbia (83.1%) [5]. In the current cohort, the median number of pDDIs (median = 8.50, range 2–19) per patient was higher than the range of median number of pDDIs (2–4.8) reported by studies conducted elsewhere [3, 7, 14, 15]. The inclusion of patients from CICU, where higher frequency of pDDIs is expected, and following the CVD patients throughout their hospital stay, which may increase the risks of pDDIs from multiple drug exposures could be some of the possible reasons for comparatively high frequency of pDDIs in the present cohort. Moreover, the other possible reasons for discrepancy in pDDIs, in different studies could be the use of different screening tools and prescribing patterns, the nature of drugs and use of narrow therapeutic index drugs, the different study designs, settings and subjects included, and the availability of clinical pharmacists at the study settings [3–8, 14–16]. Nevertheless, previously reported somewhat similar prevalence of pDDIs in CVD patients (range 91.6–96.5%) from Pakistan [3, 6] signifies the irrational prescribing practices in these patients at the study sites, and advocates for the inclusion of DDIs screening tools in the country’s healthcare settings particularly in the secondary and tertiary care hospitals. We found that 74.06% pDDIs were of moderate and 17.33% of major severity. Similarly, previous studies from Pakistan and elsewhere have also reported the moderate pDDIs as the most prevalent type in CVD patients. A study conducted in the cardiology department of Ayub Teaching Hospital (ATH) Abbottabad, Pakistan indicated 55% of moderate pDDIs [3]. However, the prevalence of severe pDDIs (45%) in their study was relatively higher than ours [3]. Likewise, another study from ATH Abbottabad, reported 56.3% moderate and 25.4% major pDDIs [14].

On the basis of Lexicomp’s risk classification, Class D and X pDDIs are assigned high risks which, respectively, need therapy modification and avoiding the combinations. List of interacting pairs involved in class D and X pDDIs given in Table 3 will be helpful for the healthcare providers to identify, prevent and manage them at the current study sites. In our study, the combination of anti-platelets and anti-coagulants accounted for 77.1% (449/582) of class D pDDIs. This finding was in line with reports from elsewhere [6, 8, 15]. Although, electronic databases are useful in recognizing pDDIs and can be used to make a clinical decision regarding modification of the treatment regimen or discontinuation of the interacting drug pairs [6]. However, modifying the treatment regimen solely based on the information provided by these databases without assessing the patient’s specific requirements may lead to irrational decisions, thereby complicating the patient’s condition. For instance, it is likely that many doctors prescribe anti-platelets and anti-coagulants in combination with balancing the risks of hemorrhage against the risk of thromboembolism which may occur in the absence of the combination therapy [15]. Nevertheless, the benefits of such combinations not always outweigh their risks; therefore, decisions regarding such combinations must always be tailored to suit requirements of an individual patient [15]. Furthermore, there is a need for proper monitoring of prothrombin time (PT), activated partial thromboplastin time (aPTT) and international normalized ratio (INR) in patients receiving these combinations. In our study, the second most common interacting pair of class D pDDIs was the concomitant use of clopidogrel with proton pump inhibitors (PPIs), i.e., omeprazole and esomeprazole, which accounted for 17.18% (100/582) of class D pDDIs. Being a prodrug, clopidogrel is converted to its active metabolite by drug metabolizing enzymes in two sequential oxidative steps [27, 28]. It has been reported that CYP2C19 plays significant role in both oxidative steps while, CYP3A4 contributes substantially to the second oxidative step [27, 28]. In order to reduce the gastrointestinal adverse effects of clopidogrel, PPIs are often co-prescribed with it, especially in those patients who are on dual anti-platelet therapy and at high risk of gastrointestinal bleeding [29, 30]. It has been shown that omeprazole and esomeprazole are potent inhibitors of CYP2C19 [31, 32]. However, in the published literature, there are conflicting reports regarding cardiovascular effects of the concomitant use of PPIs with clopidogrel. Some studies have reported that concomitant use of PPIs reduces the anti-platelet effect of clopidogrel and leads to major adverse cardiac events [33–36], while others have documented no clinical significant cardiovascular interaction between PPIs and clopidogrel [37, 38]. On the basis of available evidence, Food and Drug Administration of USA, Medicines and Healthcare Products Regulatory Agency of United Kingdom and European Medicines Agency discourage the use of omeprazole and esomeprazole in patients taking clopidogrel [39]. Although, there is no sufficient evidence about which of the PPIs least likely interacts with clopidogrel, but the available data advocates that pantoprazole, lansoprazole and rabeprazole least likely interact with clopidogrel and are suitable alternatives of omeprazole and esomeprazole [39]. The concomitant use of albuterol and carvedilol accounted for 50% (15/30) of class X pDDIs. Because of antagonistic actions, this interaction may reduce the benefits of both medications, particularly the bronchodilatory effect of albuterol [40]. It can be avoided by prescribing cardioselective β-blocker, i.e., bisoprolol which has 14-fold higher affinity for β1 than β2 receptors and is well tolerated in cardiopulmonary patients [41]. As class D and X pDDIs are associated with adverse outcomes and healthcare providers should be watchful about their incidence, effects and management, we grouped them together and analyzed the data for finding the factors associated with their presence. The results of multivariate analysis revealed that prevalence of class D and/or X pDDIs was significantly lower in those patients who suffered from CVD other than myocardial infarction (STEMI and NSTEMI) and significantly higher in those who received > 12 drugs. We found significantly high prevalence of class D and/or X pDDIs in MI patients. It is in line with a study conducted elsewhere [5] and might be explained by the fact that these patients take the narrow therapeutic index anti-platelets and anti-coagulants, which were the most commonly interacting drugs in class D pDDIs. The present finding of a statistically significant positive association between increased number of drugs prescribed (> 12) and higher incidence of class D and/or X pDDIs is consistent with studies conducted in Pakistan [3, 6], Serbia [5], Ethiopia [7] and Switzerland [42], and complements the widely reported finding of polypharmacy as a risk factor for the incidence pDDIs. In contrast to other studies [3, 7, 42], we did not find any association between the patient’s age and presence of class D and/or X pDDIs.

### Study limitation

The findings of the current study should be interpreted with the major limitations of enrolling patients with convenient sampling method, limited number of patients and lack of information about adverse events plausibly caused by DDIs. We also did not evaluate the mechanism and time of onset of pDDIs.

## Conclusion

The current study found a high prevalence of pDDIs in CVD patients who received treatment at the two major tertiary care teaching hospitals of Balochistan. The majority of pDDIs were of moderate severity. The prevalence of class D and/or X pDDIs was significantly high in those patients who suffered from MI and took > 12 drugs. Anti-platelets, anti-coagulants, PPIs, β-agonists and antagonists were involved in class D and/or X pDDIs. Clinical pharmacists are specialists in pharmacotherapy and identifying, preventing and resolving drug therapy problems (DTPs) including DDIs. Their inclusion as full member of the multidisciplinary team can improve the patients’ outcomes by developing pharmaceutical care. The provision of pDDIs screening tools, clinical decision support software, physicians’ alert systems, the development and implementation of precautionary guidelines, the regulation of appointing clinical pharmacists in healthcare settings and integrating them as full members of the healthcare teams have significantly contributed in reducing DTPs, improving patients’ outcomes and reducing healthcare costs [43–48]. Therefore, in developing countries like Pakistan, a mix of the above-mentioned strategies with the availability of well-trained clinical pharmacist(s) on the wards are suggested to reduce the incidence of pDDIs, improve patients’ outcomes and reduce healthcare costs. Moreover, the baseline information obtained through this study can be used in future for designing interventions to reduce the frequency of pDDIs and promotes rational prescribing practices in these patients.

## Abbreviations

PDDIs: Potential drug–drug interactions
DDIs: Drug–drug interactions
CVD: Cardiovascular disease
ADEs: Adverse drug events
USA: United States of America
MI: Myocardial infarction
HTN: Hypertension
DM: Diabetes mellitus

## References

[1] Forouzanfar MH, Liu P, Roth GA, Ng M, Biryukov S, Marczak L (2017). Global burden of hypertension and systolic blood pressure of at least 110 to 115 mm Hg, 1990–2015. JAMA.

[2] Isetts BJ, Schondelmeyer SW, Artz MB, Lenarz LA, Heaton AH, Wadd WB (2008). Clinical and economic outcomes of medication therapy management services: the Minnesota experience. J Am Pharm Assoc.

[3] Murtaza G, Khan MYG, Azhar S, Khan SA, Khan TM (2016). Assessment of potential drug–drug interactions and its associated factors in the hospitalized cardiac patients. Saudi Pharm J.

[4] Kovačević M, Vezmar Kovačević S, Radovanović S, Stevanović P, Miljković B (2019). Adverse drug reactions caused by drug–drug interactions in cardiovascular disease patients: introduction of a simple prediction tool using electronic screening database items. Curr Med Res and Opin.

[5] Kovačević M, Vezmar Kovačević S, Miljković B, Radovanović S, Stevanović P (2017). The prevalence and preventability of potentially relevant drug–drug interactions in patients admitted for cardiovascular diseases: a cross-sectional study. Int J Clin Pract.

[6] Shakeel F, Khan JA, Aamir M, Hannan PA, Zehra S, Ullah I (2018). Risk of potential drug–drug interactions in the cardiac intensive care units: a comparative analysis between 2 tertiary care hospitals. Saudi Med J.

[7] Diksis N, Melaku T, Assefa D, Tesfaye A (2019). Potential drug–drug interactions and associated factors among hospitalized cardiac patients at Jimma University Medical Center, Southwest Ethiopia. SAGE Open Med.

[8] Sharma S, Chhetri HP, Alam K (2014). A study of potential drug–drug interactions among hospitalized cardiac patients in a teaching hospital in Western Nepal. Indian J Pharmacol.

[9] Abdela OA, Bhagavathula AS, Getachew H, Kelifa Y (2016). Risk factors for developing drug-related problems in patients with cardiovascular diseases attending Gondar University Hospital. Ethiopia J Pharm Bioallied Sci.

[10] Ernst FR, Grizzle AJ (2001). Drug-related morbidity and mortality: updating the cost-of-illness model. J Am Pharm Assoc.

[11] Krähenbühl-Melcher A, Schlienger R, Lampert M, Haschke M, Drewe J, Krähenbühl S (2007). Drug-related problems in hospitals. Drug Saf.

[12] Janchawee B, Wongpoowarak W, Owatranporn T, Chongsuvivatwong V (2005). Pharmacoepidemiologic study of potential drug interactions in outpatients of a university hospital in Thailand. J Clin Pharm Ther.

[13] Zheng WY, Richardson L, Li L, Day R, Westbrook J, Baysari M (2018). Drug–drug interactions and their harmful effects in hospitalised patients: a systematic review and meta-analysis. Eur J ClinPharmacol.

[14] Ismail M, Iqbal Z, Khattak MB, Khan MI, Javaid A, Khan TM (2012). Potential drug–drug interactions in cardiology ward of a teaching hospital. Health Med.

[15] Patel VK, Acharya LD, Rajakannan T, Surulivelrajan M, Guddattu V, Padmakumar R (2011). Potential drug interactions in patients admitted to cardiology wards of a south Indian teaching hospital. Austaras Med J.

[16] Namazi S, Moosavi N (2012). The evaluation and management of drug–drug interactions in patients on cardiovascular and cardiosurgery wards in Namazi and Shahid Faghihi hospitals, Iran, Shiraz. Res Pharm Sci.

[17] Zubair F, Nawaz SK, Nawaz A, Nangyal H, Amjad N, Khan MS (2018). Prevalence of cardiovascular diseases in Punjab, Pakistan: a cross-sectional study. J Public Health.

[18] Javaid F, Hanif S, Syed MA, Jawad S, Afzal S, Malik R (2017). Incidence of potential drug–drug interactions in patients with cardiovascular complications due to the trend of polypharmacy prescription in Pakistan. Lat Am J Pharm.

[19] Abdullah A, Ahmad N, Atif M, Khan S, Wahid A, Ahmad I (2020). Treatment outcomes of childhood tuberculosis in three districts of Balochistan, Pakistan: findings from a retrospective cohort study. J Trop Pediatr.

[20] The Express Tribune. Civil Hospital, Quetta: Lack of facilities paint dismal picture of health sector. The Express Tribune.https://tribune.com.pk/story/1620711/civil-hospital-quetta-lack-facilities-paint-dismal-picture-health-sector (22 January, 2021, date last accessed)

[21] Daniel, W. W. (1995). *Biostatistics: a foundation for analysis in the health sciences*. New York-Chichester-Brisbane-Toronto-Singapore

[22] Lexicomp Online^®^, LexiInteract. Hudson, Ohio: Lexi‐Comp, Inc. (Accessed on 20 December, 2020).

[23] Kheshti R, Aalipour M, Namazi S (2016). A comparison of five common drug–drug interaction software programs regarding accuracy and comprehensiveness. J Res Pharm Pract.

[24] Nusair MB, Al-Azzam SI, Arabyat RM, Amawi HA, Alzoubi KH, Rabah AA (2020). The prevalence and severity of potential drug–drug interactions among adult polypharmacy patients at outpatient clinics in Jordan. Saudi Pharm J.

[25] Štuhec M, Potočin I, Stepan D, Ušaj L, Šter MP, Beović B (2019). Potential drug interactions with antibacterials in long-term care facilities analyzed by two interaction checkers. InterJ Clin Pharm.

[26] Mosca L, Barrett-Connor E, Kass WN (2011). Sex/gender differences in cardiovascular disease prevention: what a difference a decade makes. Circulation.

[27] Sangkuhl K, Klein TE, Altman RB (2010). Clopidogrel pathway. Pharmacogenet Genomics.

[28] Kazui M, Nishiya Y, Ishizuka T, Hagihara K, Farid NA, Okazaki O (2010). Identification of the human cytochrome P450 enzymes involved in the two oxidative steps in the bioactivation of clopidogrel to its pharmacologically active metabolite. Drug Metabol Dispos.

[29] Bundhun PK, Teeluck AR, Bhurtu A, Huang W-Q (2017). Is the concomitant use of clopidogrel and Proton Pump Inhibitors still associated with increased adverse cardiovascular outcomes following coronary angioplasty? A systematic review and meta-analysis of recently published studies (2012–2016). BMC Cardiovasc Disord.

[30] Members WC, Abraham NS, Hlatky MA, Antman EM, Bhatt DL, Bjorkman DJ (2010). ACCF/ACG/AHA 2010 Expert Consensus Document on the concomitant use of proton pump inhibitors and thienopyridines: a focused update of the ACCF/ACG/AHA 2008 expert consensus document on reducing the gastrointestinal risks of antiplatelet therapy and NSAID use: a report of the American College of Cardiology Foundation Task Force on Expert Consensus Documents. Circulation.

[31] Ogilvie BW, Yerino P, Kazmi F, Buckley DB, Rostami-Hodjegan A, Paris BL (2011). The proton pump inhibitor, omeprazole, but not lansoprazole or pantoprazole, is a metabolism-dependent inhibitor of CYP2C19: implications for coadministration with clopidogrel. Drug Metabol Dispos.

[32] Zvyaga T, Chang S-Y, Chen C, Yang Z, Vuppugalla R, Hurley J (2012). Evaluation of six proton pump inhibitors as inhibitors of various human cytochromes P450: focus on cytochrome P450 2C19. Drug Metabol Dispos.

[33] Arbel Y, Birati EY, Finkelstein A, Halkin A, Kletzel H, Abramowitz Y (2013). Platelet inhibitory effect of clopidogrel in patients treated with omeprazole, pantoprazole, and famotidine: a prospective, randomized, crossover study. Clin Cardiol.

[34] Serbin MA, Guzauskas GF, Veenstra DL (2016). Clopidogrel-proton pump inhibitor drug–drug interaction and risk of adverse clinical outcomes among PCI-treated ACS patients: a meta-analysis. J Manag Care Spec Pharm.

[35] Hulot J-S, Collet J-P, Silvain J, Pena A, Bellemain-Appaix A, Barthélémy O (2010). Cardiovascular risk in clopidogrel-treated patients according to cytochrome P450 2C19* 2 loss-of-function allele or proton pump inhibitor coadministration: a systematic meta-analysis. J Am Coll Cardiol.

[36] Goodman SG, Clare R, Pieper KS, Nicolau JC, Storey RF, Cantor WJ (2012). Association of proton pump inhibitor use on cardiovascular outcomes with clopidogrel and ticagrelor: insights from the platelet inhibition and patient outcomes trial. Circulation.

[37] Bhatt DL, Cryer BL, Contant CF, Cohen M, Lanas A, Schnitzer TJ (2010). Clopidogrel with or without omeprazole in coronary artery disease. N Eng J Med.

[38] O'Donoghue ML, Braunwald E, Antman EM, Murphy SA, Bates ER, Rozenman Y (2009). Pharmacodynamic effect and clinical efficacy of clopidogrel and prasugrel with or without a proton-pump inhibitor: an analysis of two randomised trials. Lancet.

[39] Tim Meadows, Medicines Information Pharmacist, Midlands and East Medicines Advice Service . Do proton pump inhibitors reduce the clinical efficacy of clopidogrel? 2019. https://www.sps.nhs.uk/wp-content/uploads/2019/07/UKMI_QA_Clopidogrel-PPI_July_2019.pdf (22 January 2021, date last accessed)

[40] Egred M, Shaw S, Mohammad B, Waitt P, Rodrigues E (2005). Under-use of beta-blockers in patients with ischaemic heart disease and concomitant chronic obstructive pulmonary disease. QJM.

[41] Jabbal S, Anderson W, Short P, Morrison A, Manoharan A, Lipworth BJ (2017). Cardiopulmonary interactions with beta-blockers and inhaled therapy in COPD. QJM.

[42] Egger SS, Bravo AER, Hess L, Schlienger RG, Krähenbühl S (2007). Age-related differences in the prevalence of potential drug–drug interactions in ambulatory dyslipidaemic patients treated with statins. Drugs Aging.

[43] Lopez-Martin C, Siles MG, Alcaide-Garcia J, Felipe VF (2014). Role of clinical pharmacists to prevent drug interactions in cancer outpatients: a single-centre experience. Int J Clin Pharm.

[44] Graabæk T, Kjeldsen LJ (2013). Medication reviews by clinical pharmacists at hospitals lead to improved patient outcomes: a systematic review. Basic Clin Pharmacol Toxicol.

[45] Georgiev KD, Hvarchanova N, Georgieva M, Kanazirev B (2019). The role of the clinical pharmacist in the prevention of potential drug interactions in geriatric heart failure patients. Int J Clin Pharm.

[46] Tannenbaum C, Sheehan NL (2014). Understanding and preventing drug–drug and drug–gene interactions. Expert Rev Clin Pharmacol.

[47] AlRuthia Y, Alkofide H, Alosaimi FD, Sales I, Alnasser A, Aldahash A (2019). Drug–drug interactions and pharmacists’ interventions among psychiatric patients in outpatient clinics of a teaching hospital in Saudi Arabia. Saudi Pharm J.

[48] Moura CS, Prado NM, Belo NO, Acurcio FA (2012). Evaluation of drug–drug interaction screening software combined with pharmacist intervention. Int J Clin Pharm.

